# CMR Manifestations, Influencing Factors and Molecular Mechanism of Myocarditis Induced by COVID-19 Mrna Vaccine

**DOI:** 10.31083/j.rcm2310339

**Published:** 2022-10-11

**Authors:** Chao-Fei Ba, Bing-Hua Chen, Li-Shi Shao, Ya Zhang, Chen Shi, Lian-Ming Wu, Jian-Rong Xu

**Affiliations:** ^1^Department of Radiology, The Second Affiliated Hospital of Kunming Medical University, 650500 Kunming, Yunnan, China; ^2^Department of Radiology, RenJi Hospital, School of Medicine, Shanghai Jiao Tong University, 200127 Shanghai, China; ^3^Department of Radiology, The Third Affiliated Hospital of Kunming Medical University, Yunnan Cancer Hospital, Xishan District, 650118 Kunming, Yunnan, China

**Keywords:** COVID-19, mRNA vaccines, myocarditis, CMR, pathogenesis

## Abstract

Although immunization with the 2019 coronavirus disease (COVID-19) mRNA vaccine is considered to be an effective measure to reduce the number of serious cases or deaths associated with COVID-19, rare cases of cardiac complications have been reported in the literature, encompassing acute myocardial injury, arrhythmia, vasculitis, endothelial dysfunction, thrombotic myocardial infarction and myocarditis. Interestingly, patients diagnosed with myocarditis after receiving the COVID-19 mRNA vaccine exhibit abnormal cardiac magnetic resonance (CMR) findings, suggesting CMR can be a valuable non-invasive diagnostic tool. In populations immunized with the COVID-19 mRNA vaccine, the risk in teenagers and young men is significantly higher. Myocardial injury in male patients is mainly myocarditis, while in female patients, myocarditis and pericardial effusion are predominantly found. Generally, the symptoms of myocarditis are relatively mild and complete recovery can be achieved. Moreover, the incidence rate associated with the second dose is significantly higher than with the first or third dose. This article brings together the latest evidence on CMR characteristics, influencing factors and pathogenesis of myocarditis caused by the COVID-19 mRNA vaccine. At the same time, we make recommendations for populations requiring immunization with the COVID-19 mRNA vaccine.

## 1. Introduction

As of June 2022, more than 530 million patients with the 2019 coronavirus disease (COVID-19) novel coronavirus have been confirmed worldwide, and the death toll has exceeded 6 million [1]. Mahmood *et al*. [2] reported that treatment with Ramdesivir and convalescent plasma (CP) is the best treatment to combat COVID-19. COVID-19 mRNA vaccine has a positive effect on preventing coronavirus infection. Dagan *et al*. [3] reported that the study of large-scale COVID-19 mRNA vaccination in Israel showed that COVID-19 mRNA vaccine could effectively prevent COVID-19 infection and reduce COVID-19 severe patients [3, 4, 5]. However, the COVID-19 mRNA vaccine has also been associated with an increased incidence of relatively rare diseases, such as myocardial injury, myocarditis thrombosis, tubulitis, macrovasculitis and Kawasaki disease [5, 6, 7, 8, 9, 10, 11, 12, 13, 14]. It has been established that the COVID-19 mRNA vaccine produces antibodies to S protein through mRNA and membrane s glycoprotein, prevents the binding of S protein with angiotensin converting enzyme2 (ACE2), and produces cellular immunity and humoral immunity, which eventually leads to myocarditis [15]. COVID-19 mRNA vaccine-related myocardial injury has been widely reported, and cardiac magnetic resonance cardiac magnetic resonance (CMR) is an important diagnostic tool for evaluating myocardial structural and functional changes [16]. The American College of Cardiology and the Cardiovascular Magnetic Resonance Association advocates that CMR is a valuable diagnostic tool for COVID-19 patients with incomplete evidence of myocardial tissue composition, myocardial injury and cardiac function decline [16, 17]. Shiyovich *et al*. [18] showed that CMR has a high diagnostic performance in diagnosing and evaluating myocarditis caused by COVID-19 vaccine treatment, especially in patients with good ejection function; CMR imaging findings are consistent with “typical myocarditis”. This review sought to provide a comprehensive overview of the imaging characteristics of CMR in the diagnosis and evaluation of patients with myocarditis caused by COVID-19 mRNA vaccine treatment and analyze the influencing factors and potential pathogenesis of myocarditis. Finally, we made some suggestions for immunization with the COVID-19 mRNA vaccine.

## 2. Background: Myocarditis Injury Caused by COVID-19 Mrna Vaccine

### 2.1 Manifestations of Myocarditis Caused by COVID-19 Mrna Vaccine

Overwhelming evidence substantiates that the immunogenicity of the COVID-19 mRNA vaccine can trigger many rare cardiovascular and blood disease reactions, including myocarditis, pericardial effusion, myocardial infarction, atypical Kawasaki disease, arterial thrombosis, cutaneous small vessel vasculitis, large vessel vasculitis, etc. [7, 8, 9, 10, 11, 12, 14]. In a study on 27 patients with cardiac inflammation caused by COVID-19 vaccine, most complained of chest pain, palpitations, joint pain and dyspnea, exhibiting elevated cardiac troponin I (HS cTnI) levels, and 77.8% of patients had ST-segment elevation or T wave inversion in electrocardiogram (ECG) (Table 1a,1b, Ref. [5, 19, 20, 21, 22, 23, 24, 25, 26, 27, 28, 29, 30, 31, 32, 33, 34, 35, 36, 37]). Amir *et al*. [20] retrospectively collected 15 cases of myocarditis related to the BNT162b2 mRNA COVID-19 vaccine in five major children’s medical centers in Israel. The majority of patients were male, exhibiting symptoms and signs of myocardial involvement (such as chest pain and arrhythmia), 93.3% of patients had elevated troponin levels, 13.3% of patients had pericardial effusion, 20% of patients had ventricular dysfunction, and 86.7% of patients had nonspecific ST/T changes in ECG [20]. Puchalski *et al*. [21] retrospectively analyzed five adolescents with body mass index (BMI) values of 24.8 to 30 (4 obese and 1 overweight) vaccinated with Pfizer mRNA vaccine, which exhibited retrosternal chest pain (n = 5), elevated body temperature (n = 4), diarrhea and shoulder pain (n = 1), and dry cough (n = 1). Troponin levels were significantly increased in all cases and decreased rapidly a few days later. Echocardiography showed that the left ventricular ejection fraction ranged from 61 to 72% [21]. Six cases of myocarditis were reported out of nearly 200000 citizens vaccinated with the mRNA COVID-19 vaccine in Italy. All six patients were hospitalized due to fever and elevated troponin and were treated with colchicine and ibuprofen. One patient exhibited atrial tachycardia, and another showed right ventricular involvement. Only a female patient was diagnosed with myocarditis and pericardial effusion. The median high-sensitivity troponin and C-reactive protein (CRP) levels in these 6 patients were 2373 ng/mL and 4 ± 1.8 mg/L [22]. The University of Ulm in Germany reported four cases diagnosed with pericarditis or myocarditis after mRNA vaccination after a mean duration of 7.5 ± 6.5 days, exhibiting chest and back tingling (n = 4), fever (n = 2), abnormal ECG findings (n = 2), and increased high-sensitivity troponin T (hs-TnT) (n = 4). Two patients underwent endomyocardial biopsy and were diagnosed with non-giant cell myocarditis [23]. In the United States, where nearly 10 million people were immunized with the COVID-19 mRNA vaccine in 2021, 1626 cases of myocarditis have been reported with a median age of 21 years (16–31 years) and the median duration of symptoms of 2 days (1–3 days), consisting predominantly of males (82%), mainly adolescents [38]. The symptoms of myocarditis caused by the COVID-19 vaccine are generally mild and do not need hospitalization, but some patients will have heart failure and need heart transplantation, and death may occur in serious cases [38]. To sum up, myocarditis is a rare complication of immunization with the COVID-19 mRNA vaccine, often presenting with chest pain, fever, diarrhea, dyspnea and other symptoms. Generally, the disease is mild and can be generally cured after hospitalization.

**Table 1a.** **Study on CMR of myocarditis induced by novel coronavirus vaccine**.

First Author (Ref. #) Study Design	Country	Number of vaccinations	Number of Cases	Men	Age, ${\mathrm{ya}}^{a}$	Type of vaccination	COVID-19 vaccine doses prior to symptom onset	Time (days) from vaccine inoculation to symptoms	Patient characteristics during acute myocarditis (Clinical manifestation and laboratory examination)	Patient characteristics during the postacute stage
Mohammadi *et al*. [36] Retrospective observational study	Iran	No symptoms	1	1	20	AstraZeneca	3	4	Severe chest pain, Troponin I = 3.34	No symptoms
Dedda *et al*. [19] Retrospective observationalstudy	Europe	No symptoms	27	25/27	36.6 ± 16.8	Pfizer/BioNTech/ Moderna/AstraZeneca	1 (n = 27), 2 (n = 15)	n = 22/27; average 8 ± 9 days (range 0–10) days	Chest pain (n = 25), palpitations (n = 10), arthralgias and myalgias (n = 9), and dyspnea (n = 7), (n = 27) cases (HS cTnT) or (HS cTnI) were elevated	Short-term follow-up from presentation was uneventful for 25/27 patients (median = 20 days; range = 2–82 days) and unavailable in two cases.
Bae *et al*. [24] Retrospective observational study	Korea	No symptoms	1	0	38	mRNA1273 (Moderna)	1	4	Chest pain, mild dyspnea, and sweating, CK-MB, ng/mL (≤4.94), Troponin T, ng/mL (≤0.014)	Not reported
Amir *et al*. [20] Retrospective observational study	Israe	224000	15	15	17 ± 1 (median 17.2, range 14.9–19)	BNT162b2	2 (n = 14/15), 3 (n = 1/15)	4.4 ± 6.7 (median 3, range 0–28) days	Clinical manifestation Not reported, (14/15) patients had elevated troponin T levels	After 6 months, clinical symptoms were resolved in all patients, and one patient exhibited mild pericardial effusion. Individuals with preexisting CAD or myocarditis had abnormal ECG findings
Oka *et al*. [33] Retrospective observational study	Japan	No symptoms	1	1	50	BNT162b2	2	10	Syncope and resting chest pain; ST-segment elevation on ECG and significantly increased Cardiac troponin I	At 2 weeks after discharge, syncope, heart failure, ECG atrioventricular block, echocardiographic LVEF was 60%, and cardiac troponin I level increased slightly.
Christophe *et al*. [31] Retrospective observational study	Switzerland	93968	3	3	28.7 ± 14.2	mRNA-1273/BNT162b2	2 (n = 3)	2.3 ± 0.6	All hospitalized (100%, n = 3) patients had mild to moderate symptoms On admission, 100% (3/3) patients had troponin elevation, and 100% (n = 3) had ECG abnormalities	Not reported
Das *et al*. [32] Retrospective observational study	The United Arab Emirates	No symptoms	1	1	27	Pfizer/BioNTech	2	3	severe chest discomfort, patients had troponin elevation, ECG abnormalities	Not reported
Ansari *et al*. [37] Retrospective observational study	Germany	No symptoms	1	1	23	mRNA1273 (Moderna)	2	1	On admission, angina pectoris, the ECG was abnormal, the symptoms were serious, and the level of high-sensitivity troponin I increased	Patient asymptomatic
Nunn *et al*. [23] Retrospective observational study	Germany	No symptoms	4	3	29.5 ± 13.2	Pfizer/BioNTech	2 (n = 3/4)	7.5 ± 6.5	On admission, 75% (3/4) of patients had mild symptoms, and 125% (1/4) had moderate to severe symptoms. 4 patients had troponin elevation	Not reported
Puchalski *et al*. [21] Retrospective observational study	Poland	No symptoms	5	5	16.6 ± 0.9	Pfizer/BioNTech	1 (n = 2/5), 2 (n = 3/5)	6.4 ± 9.3	100% (5/5) of patients chest pain, 100% (5/5) of patients Increased troponin levels	Three months later (1 patient with a follow-up appointment postponed for one month due to moderate infectious symptoms), 1 patient reported a single episode of sharp chest pain lasting for a few seconds
Frustaci *et al*. [25] Retrospective observational study	Italy	100000	3	2	56.3 ± 19.8	BNT162b2	2 (n = 3/3)	Not reported	100% (n = 3/3) patients chest pain , beyond elevation of troponin I (3.5 ± 0.2 mcg/L ± n.v 0.1 ± 0.14 mcg/L) was unremarkable.	Not reported
Shaw *et al*. [34] Retrospective observational study	USA	No symptoms	4	2	22.0 ± 6.9	Pfizer/Moderna	1 (n = 2/4), 2 (2/4)	8.8 ± 10.9	100% (n = 4/4) had chest pain and elevated troponin I (n = 4/4)	Not reported
Manfredi *et al*. [22] Retrospective observational study	Italy	231989	6	4	17.5 ± 3.9	Pfizer/BioNTech and Moderna	2 (n = 6/6)	Not reported	100% (n = 6/6) fever ,The median high-sensitive Troponin-I (Hs-TnI) was 2373 (Q1, Q3: 576, 8123) ng/mL	3.0 ± 0.5 months after discharge, all patients were asymptomatic
Gomes *et al*. [35] Retrospective observational study	Portugal	No symptoms	1	1	32	SARS-CoV-2 mRNA	2	2	Syncope and chest pain, Myocardial biomarkers (high-sensitivity cardiac troponin T 834 ng/L and NT proBNP 433 mg/mL) increased	After discharge, epicardial involvement during late gadolinium enhancement was significantly improved, and T1 and T2 normalized
Meyer-Szary *et al*. [28] Retrospective observational study	Poland	No symptoms	3	3	19.3 ± 8.7	Spikevax Moderna Comiranty	2 (n = 3/3)	1.7 ± 0.6	Elevated troponin I, 100% (n = 3/3) Severe stinging chest pain	Not reported
Kravchenko *et al*. [27] Retrospective observational study	Germany	No symptoms	20	12	28 ± 12	Pfizer/BioNTech or Moderna	1 (n = 5/20), 2 (n = 15/20)	1.1 ± 1.2 (LLC-positive) 2018 Lake Louise criteria (LLC)	85% of patients (17/20) had chest pain, 55% of patients (11/20) had dyspnea, and 10% of patients (2/20) had a fever, troponin T levels (3938 ± 5850 ng/L vs. 9 ± 11 ng/L; *p *< 001)	Not reported
Patel *et al*. [30] Retrospective observational study	USA	No symptoms	5	5	24.6 ± 7.3	Pfizer/Moderna	1 (n = 1/5), 2 (n = 4/5)	1.8 ± 0.4	Chest pain and elevated troponin I in 100% (n = 5/5), dyspnea in 60% (n = 3/5)	Not reported
Chelala *et al*. [29] Retrospective observational study	USA	No symptoms	5	5	17.2 ± 1.0	Pfizer-BioNTech/Moderna	2 (n = 5/5)	3.6 ± 0.6	Chest pain and elevated troponin I in 100% (n = 5/5) of patients and abnormal ECG in 40% (n = 2/5)	Not reported
Choi *et al*. [26] Retrospective observational study	Korea	No symptoms	1	1	22	BNT162b2 mRNA	1	22	Chest pain, ventricular fibrillation, laboratory examination not reported	Not reported

^a^ SD or median (interquartile range); Ya, Year; CRM, Cardiac magnetic resonance; LGE, late gadolinium enhancement; ECG, electrocardiogram; CAD, Coronary Heart Disease; TnT, troponin T; HS cTnI, High-sensitivity troponin I; HS cTnT, High-sensitivity troponin T; CK-MB, creatine kinase isoenzyme; LLC, Lake Louise criteria; LV, Left ventricle; RV, Right ventricle; STIR, short time inversion recovery; T2WI, T2 weighted imaging; T1WI, T1 weighted imaging; LVEF, Left ventricular ejection fraction; EF, Ejection fraction; LVEDV, Left ventricular end diastolic volume; LVEDVI, Left ventricular end diastolic volume index; RVEDV, Right ventricular end diastolic volume index; IVSD, interventricular septal diameter; ECV, extracellular volume; GRS, global radial strain; GCS, global circumferential strain; GLS, global longitudinal strain; NT proBNP, N-terminal pro b-type natriuretic peptide.

**Table 1b.** **Study on CMR of myocarditis induced by novel coronavirus vaccine**.

First Author (Ref. #) Study Design	Histopathological evidence	LGE	Myocardial parametric mapping	LV/RV structure and function, pericardial disease
Mohammadi *et al*. [36] Retrospective observational study	Not reported	subepicardial/mid-wall enhancement in the basal inferior and anterior apical segments of LV	subepicardial/mid-wall enhancement in the basal inferior and anterior apical segments of LV STIR T2WI (increased signal intensity in the inferior basal segment; myocardial inflammation	Echocardiography and cardiac troponin were normal, without any symptoms
Dedda *et al*. [19] Retrospective observational study	Not reported	85% (n = 23) patients had LGE and T2 enhancement	CMR revealed typical mid-subepicardial nonischemic late gadolinium enhancement (LGE) in 23 cases and matched positively with CMR T2 criteria of myocarditis.	Not reported
Bae *et al*. [24] Retrospective observational study	A small number of eosinophils, T lymphocytes and macrophages	The patient had LGE and elevated T2	LGE in the left anterior inferior septum and the middle of the left anterior inferior septum. T2 left ventricular basal wall to middle wall high signal	Normal left and right ventricular functions, no regional wall motion abnormalities, normal diastolic function, normal ejection fraction of 67%, right ventricular systolic pressure of 29 mmHg
Amir *et al*. [20] Retrospective observational study	Not reported	26% (n = 4) of patients had elevated T2, and 93% (n = 14) of patients had LGE.	26% (n = 4) of patients had elevated T2, 93% (n = 14) of patients had LGE. The subepicardial layer in the mid-myocardial region of the left ventricle was involved in 100% (n = 15) of patients	Not reported
Oka *et al*. [33] Retrospective observational study	A small number of eosinophils, T lymphocytes and macrophages	Elevated T2 and positive LGE	Patient had LGE and elevated T2	The patient was discharged 22 days after admission; the echocardiography showed recovery of the LVEF to 60%
Christophe *et al*. [31] Retrospective observational study	Not reported	100% (n = 3) of patients had positive LGE	3 patients had LGE	Not reported
Das *et al*. [32] Retrospective observational study	Not reported	The patient had LGE and elevated T2	Patient had both elevated T2 and positive LGE in the LV’s basal and mid-anterolateral, posterolateral, and inferoseptal segments.	Patient baseline values for LV GLS (−14.55), RV GLS (−15.8), and RVCS were all considerably lower (−6.88).
Ansari *et al*. [37] Retrospective observational study	Not reported	The patient had LGE and elevated T1	Native T1 maps revealed a diffuse increase in relaxation times in all myocardial segments [1,344 ± 74 ms; normal range < 1,228 ms (1,181 ± 47 ms) for this 3T machine]	A follow-up CMR performed after 3 months revealed a markedly improved LVEF (57%)
Nunn *et al*. [23] Retrospective observational study	Acellular myocarditis	50% (n = 2) of patients had had hyperenhancement on their T2 and T1 sequences 100% (n = 4) of patients had LGE	4 patients had LGE, and 2 patients had elevated T2	LVEDVI was greater than 70 (81 ± 5.5) in 4 cases, and RVEDVI was greater than 60 but less than 100 (79.8 ± 11.0) in 4 cases
Puchalski *et al*. [21] Retrospective observational study	Not reported	Subepicardial, subepicardial and intraventricular LGE in segments and elevated T2.	5 patients had LGE and elevated T2.	Echocardiography %EF of 5 patients: 64, 72, 61, 62, 68
Frustaci *et al*. [25] Retrospective observational study	Strong infiltration of eosinophils	CMR showed three patients increase in both T2 and T1 myocardial, and LGE was present in the subepicardial myocardium.	3 patients had LGE and elevated T1 and T2.	Two males showed severe compromise of myocardial contractility (left ventricular ejection fraction ≤35%). The female patient exhibited a junctional rhythm on ECG
Shaw *et al*. [34] Retrospective observational study	Not reported	100% (n = 4) of patients had elevated T2. 100% (n = 4) of patients had LGE	4 patients had LGE and elevated T2. 4 patients T1 (1111 MS, 1117 ms-1137 MS, 1122 ms-1128 MS, 1172 MS are greater than the normal range 950 ms-1050 MS)	1 patient had mildly decreased systolic function (LVEF 1/4 54%), and 3 patients had a normal systolic function
Manfredi *et al*. [22] Retrospective observational study	Not reported	100% (n = 6) of patients had LGE	Myocarditis was present in males (65% (n = 4/6)) and characterized by myocardial edema (T2w hyperenhancement) and LGE in females was predominantly myopericarditis (30% (n = 2/6)).	LVEDV (68.8 ± 5.9)
Gomes *et al*. [35] Retrospective observational study	Not reported	Patient had LGE, elevated T1 and T2	The delayed enhancement of epicardial gadolinium in the middle anterior wall, lateral wall and inferior wall and the increase of natural T1 and T2	The left ventricular ejection fraction remained unchanged (58%), the segmental contractility was normal, but the overall longitudinal strain decreased slightly (–17%)
Meyer-Szary *et al*. [28] Retrospective observational study	Not reported	Patients had LGE, elevated T1 and T2	Increased T2 and T1 relaxation times in parametric mapping and a matching late gadolinium enhancement (LGE) area suggestive of irreversible damage	The LVEF (%) of the three patients were 65%, 58% and 63%, respectively
Kravchenko *et al*. [27] Retrospective observational study	Not reported	The T2 signal intensity of LLC-positive patients increased, and the incidence of LGE was higher.	Compared with the control group (1.6 ± 0.3), the T2 signal intensity ratio of LLC positive patients increased (2.0 ± 0.3, *p* = 0.012), and the incidence of LGE was higher (n = 9100%) than that of LLC negative group (n = 0 of 11 cases) and control group (n = 0 of 40 cases).	Cardiac MRI parameters (LVEF, *p* = 0.34), (LVEDV, *p* = 0.34), left ventricular diastolic (LVEDVI, *p* = 0.05), (IVSD, *p* = 0.37) (ECV, *p* = 0.23) were compared, and there was no difference between groups
Patel *et al*. [30] Retrospective observational study	Not reported	Patients had LGE, elevated T1 and T2	Late gadolinium enhancement on 100% (5/5) T1 weighted images and 60% (3/5) T2 weighted hyperintensity of myocardial edema	100% (5/5) CMR LVEF is normal
Chelala *et al*. [29] Retrospective observational study	Not reported	Patients had LGE and EGE, elevated T2	4 patients had Ege and LGE. Ege and LGE mainly affect the epicardium of the inferior wall, inferior lateral wall, etc.	20% (1/5) LVEF decreased,80% (4/5) LVEF is normal
Choi *et al*. [26] Retrospective observational study	Mainly neutrophils	Not reported (Death)	Not reported (Death)	Not reported (Death)

### 2.2 Laboratory and Histopathological Evidence of Myocarditis Caused by COVID-19 Vaccine

COVID-19 disease and COVID-19 mRNA vaccine immunization have been associated with myocarditis; however, significant heterogeneity surrounds the degree of myocardial lesions and histopathology. The following cases can support this view. COVID-19 induced myocarditis is also considered to be a relatively rare disease. Macrophage and T cell infiltrations have been documented in the dead patients with myocarditis and autopsy and the analysis of samples stained with eosin methylene blue (EMB). Myocarditis involving macrophage and T cell infiltration were also observed in the non-infectious death (control group) and COVID-19 cases. However, the infiltration degree in each condition is different, and in both cases, these findings do not represent clinically relevant myocarditis. In addition, in SARS-CoV-2 patients, myocardial tissue cells exhibit significant macrophage inflammatory infiltration, which is related to the viral lymphoid effect [39]. Kawakami performed an autopsy of the myocardium in 16 patients who died of SARS-CoV-2 infection. In one case, myocarditis with macrophage and T cell infiltration was found [39]. Bae *et al*. [24] reported that a 38-year-old female was diagnosed with myocarditis 4 days after receiving the mrna-1273 vaccine (Moderna). After ventricular septal tissue sections were harvested, hematoxylin-eosin staining and immunohistochemical staining for leukocyte common antigen (LCA) were performed. Lymphocytic infiltration was found in muscle fibers and stroma [24]. A 50-year-old man was diagnosed with myocarditis after vaccination, presenting with chest pain with tachycardia and ST-segment elevation observed on the ECG. Laboratory examination showed that cardiac troponin I was significantly increased, the left ventricular ejection fraction (LVEF) on cardiac ultrasound was 35%, and gadolinium enhancement was observed in late left ventricular imaging. The right ventricular septal endocardial myocardium was biopsied, and hematoxylin-eosin staining and immunostaining were performed. A small number of eosinophils, T lymphocytes and macrophages were found in some tissues, indicating that myocardial cells sustained inflammation and damage [40]. Nunn *et al*. [23] reported that a 31-year-old female patient with myocarditis was inoculated with the BioNTech/Pfizer vaccine 17 days ago. The hs-TnT and N-terminal pro b-type natriuretic peptide (NT-proBNP) were increased. The patient underwent a myocardial biopsy and eosin methylene blue staining. Finally, the patient was diagnosed with non-giant cell myocarditis [23]. Three patients with myocarditis caused by BNT162b2 vaccination were reported by the University of La Pienza in Rome, Italy. Compared with the control group (6.1 ± 1 u/mL) of vaccinated patients without myocarditis, the serum cationic protein level of all patients increased (23.4 ± 17 u/mL) (*p* = 0.079). The myocardial tissue was harvested for pathological section and stained with human eosinophil major basic protein (EMBP), showing the crystal degranulation of eosinophils, indicating myocardial injury and strong infiltration of eosinophils. It was found that a large number of myocardial cells died and were surrounded by eosinophils in the myocardial tissue (IHC antibody) [25]. A 22-year-old Korean male suffered from chest pain, ECG and related examinations: ventricular fibrillation, myocarditis, and finally sudden death 5 days after receiving BNT162b2 mRNA vaccine. Myocardial tissue sections were harvested and stained with hematoxylin and eosin, showing a large number of inflammatory infiltrates (mainly neutrophils) in myocardial cells. Inflammatory cells were mainly distributed in the atrium and sinoatrial node, and ventricular contraction zone cells were necrotic [26]. COVID-19-induced myocarditis is also considered to be a relatively rare disease. It has been found that macrophage and T cell infiltration can be seen in dead patients with myocarditis. Although myocarditis involving macrophage and T cell infiltration can also be seen in the non-infectious death (control group) and COVID-19 cases, the degree for each condition is different, and in both cases, these findings do not represent clinically relevant myocarditis. In addition, in SARS-CoV-2 patients, myocardial tissue cells exhibit high macrophage inflammatory infiltration, which is related to the viral lymphoid effect [39]. Kawakami *et al*. [39] performed an autopsy of the myocardium in 16 patients who died of SARS-CoV-2 infection. Myocarditis with macrophage and T cell infiltration was found in one case diagnosed with myocarditis [39]. The above findings suggest significant eosinophil infiltration in cases of myocarditis tissue caused by the COVID-19 vaccine, while macrophages are mainly present in the myocarditis associated with COVID-19 disease [26, 39, 41]. The reasons and mechanisms for the above differences remain unclear, warranting further research.

## 3. CMR Evaluation of Myocarditis Caused by the COVID-19 Mrna Vaccine

### 3.1 CMR Evaluation of Myocarditis Caused by COVID-19 Mrna Vaccine

The 2018 Lake Louise criteria (LLC) upgraded version requires that the diagnosis of Acute myocarditis (AM) must meet at least one sequence sensitive to edema (T2 weighted imaging or T2 mapping) and at least one T1 sequence (T1 mapping, ECV, myocardial delayed enhancement imaging) and be positive at the same time. T1 weighted images can reflect the difference in longitudinal relaxation of myocardial tissue and reduce the influence of transverse relaxation of other tissues. In cases of acute myocarditis, gadolinium injection can enhance the early development of the myocardium. In contrast, T2 weighted images can highlight the difference in transverse relaxation. When myocardial edema occurs, the tissue shows a strong signal, and T2 time is prolonged [42, 43, 44, 45, 46, 47]. Giulia *et al*. [48] compared the diagnostic performance of the new and old LLC in different clinical manifestations: myocardial infarction, cardiomyopathy, and arrhythmia. Using T2-weighted short-tau inversion recovery (T2w-STIR), T2 mapping, T1 weighted images, and late gadolinium enhancement (LGE), the positive rate of the new standard LLC was 58.3%, while the positive rate of the old standard LLC was 37.9% when patients had clinical manifestations of myocardial infarct, cardiomyopathy and arrhythmia. The new LLC standard significantly improves the accuracy of CMR in diagnosing acute myocarditis, especially in patients with myocarditis whose clinical manifestations are not obvious [48]. Kravchenko *et al*. [27] found that the CMR results of 20 patients with myocarditis suspected to be caused by COVID-19 mRNA vaccine immunization exhibited similar manifestations to viral myocarditis, and LLC met the diagnostic criteria. Shiyovich *et al*. [18] reported that the CMR imaging results of myocarditis patients inoculated with Pfizer BNT162b2 vaccine were inconsistent with those of the latest early diagnosis standard of LLC, but selective bias could not be ruled out. In addition, in patients clinically diagnosed with myocarditis after vaccination, the CMR imaging results were relatively mild and consistent with the performance of “classic myocarditis”. Pan *et al*. [49] reported that natural T1 has a significant advantage over LLC in evaluating the sensitivity of myocarditis. Emanuele *et al*. [19] reported the diagnosis and manifestations of nonischemic epicardial LGE and myocarditis in 23 cases of CMR. The CMR features of acute myocarditis include a high signal (nonischemic epicardium) on the short axis of T2 weighted STIR, and the results are consistent with LGE. At the same time, acute myocarditis can be confirmed on surface images T1 and T2 [19]. It has been established that cardiac magnetic resonance tissue feature tracking is more effective than traditional LLC in diagnosing myocarditis, especially in patients with good ejection function. Therefore, CMR plays a positive role in diagnosing and evaluating myocarditis caused by immunization with the 2019 coronavirus disease vaccine [18, 50].

### 3.2 CMR Performance in Patients with Myocarditis Caused by COVID-19 Vaccine Immunization during Hospitalization

The CMR manifestations of myocarditis caused by COVID-19 mRNA vaccine treatment are mainly abnormalities in the inferior epicardial wall, the inferior lateral segment of the myocardium, and the inferior wall of the myocardium [51, 52]. The following cases can support this view. Dedda *et al*. [19] reported 27 cases of myocarditis caused by the 2019 coronavirus vaccine, exhibiting chest pain (n = 25), palpitations (n = 10), myalgia (n = 9) and dyspnea (n = 7), of which 77.8% (n = 21/27) exhibited increased cardiac troponin T, 85.1% (n = 23/27) displayed non-ischemic late gadolinium enhancement under epicardium matched with CMR T2 images, and 25.9% (n = 7/27) showed pericarditis. Meyer-Szary *et al*. [28] reported three patients with myocarditis immunized with the COVID-19 RNA vaccine. CMR showed that myocardial damage mainly occurred in the lower and lower lateral segments of the myocardium, and T2 weighted stir epicardial edema signal [28]. Consistent with findings reported by Chelala *et al*. [29]. In 5 patients with acute myocarditis after immunization with the COVID-19 mRNA vaccine, CMR showed that all patients had LGE with simple epicardial enhancement (n = 4), involvement of the inferior wall or anterolateral wall (n = 5), epicardial enhancement (n = 1), and increased myocardial T2 signal intensity (n = 5) [29]. Patel *et al*. [30] reported the acute myocardium of 5 young men after receiving the COVID-19 mRNA vaccine. CMR showed that myocardial edema and LGE were mainly distributed in the bottom and middle lateral of the left ventricle, and the prognosis was good [30]. Amir *et al*. [20] retrospectively analyzed 15 patients with myocarditis induced by the BNT162b2 vaccine that were predominantly young (average age of 17 ± 1 years) and male. CMR showed edema on T2 in 90% (n = 12) of patients and pathological LGE in 90% (4/5) of patients. CMR reexamination six months after discharge found myocardial scarring in 7 out of 9 patients [20]. Christophe *et al*. [31] reported 3 patients with severe myocarditis caused by the COVID-19 RNA vaccine. During CMR, all patients exhibited LGE in the inferior epicardial or inferior wall and edema in the T2 weighted sequence [31]. Das reported a male patient with myocarditis with LVEF 50% and RVEF 46%. COVID-19 RNA vaccine, epicardial and mesangial LGE were shown in the lower and lateral segments of the left ventricle [32]. Oka *et al*. [33] reported a case in a male patient where CMR showed LGE in the lower septum. T2 weighted imaging showed left ventricular (LV) myocardial wall edema. LGE disappeared after 5 weeks, but the hyperintensity of LV whole wall persisted [33]. In conclusion, CMR of myocarditis caused by COVID-19 mRNA vaccine immunization exhibits LGE in the inferior epicardial wall, the inferior lateral segment and inferior wall of the myocardium, and most cases display edema on T2.

Most of the symptoms of myocarditis after COVID-19 mRNA vaccination were mild and the prognosis was good, but some patients had severe concurrent symptoms and sequelae. For example, Choi *et al*. [26] reported that a 22-year-old man in Korea developed chest pain 5 days after the first dose of BNT162b2 mRNA vaccine and died 7 hours later, autopsy showed that the cause of death was myocarditis [26]. Amir *et al*. [20] reported that a patient diagnosed with myocarditis after COVID-19 mRNA vaccine had pericardial effusion 6 months later. Oka *et al*. [33] reported that a Korean myocarditis patient developed syncope, heart failure and atrioventricular block 2 weeks after discharge. To sum up, myocarditis after COVID-19 mRNA vaccination needs to be paid great attention. However, it is difficult to diagnose by laboratory tests and other diagnostic methods in the early stage of the disease. CMR is sensitive and effective to discriminate early myocarditis [50].

## 4. Influencing Factors of Myocarditis Caused by COVID-19 mRNA Vaccine

Statistical analysis showed that the influencing factors of myocarditis caused by the COVID-19 mRNA vaccine include: gender, age, vaccine dose times and others [53]. In this respect, 27 cases of myocarditis caused by the COVID-19 mRNA vaccine reported by Emanuele *et al*. [19] were predominantly young (36.6 ± 16.8 years old) and male (n = 25/27) and associated with the first (n = 12/27) and second (n = 15/27) doses. Amir *et al*. [20] reported 15 patients (mean age 17 ± 1 years) with myocarditis caused by the BNT162b2 vaccine, all of whom were male (n = 15/15), associated with the second 93.3% (n = 14) and third 6.6% (n = 1) doses. Kravchenko *et al*. [27] reported that 20 patients with post-vaccine myocarditis, aged 28 ± 12 years, were predominantly male (n = 12), occurring at an average of 1.1 ± 1.2 days after vaccination. In Italy, 6 cases (predominantly male (4/6) and aged 17.5 ± 3.9 years) were hospitalized due to fever and elevated troponin after the second dose of the mRNA COVID-19 vaccine and diagnosed with myocarditis. The main manifestations in female patients were myocarditis and pericardial effusion [22]. Dedda *et al*. [19] reported 4 patients (male (n = 2)) aged 22.0 ± 6.9 years) who developed symptoms of acute myocarditis at 8.8 ± 10.9 days after the first (2/4) and second dose (2/4) of Pfizer and Moderna vaccines [34]. Puchalski *et al*. [21] reviewed 5 male patients aged 16.6 ± 0.9 years with typical myocarditis caused by the COVID-19 vaccine. The CMR diagnosis was myocarditis after the first (2/5) and second dose (3/5). Elevated troponin (1674–37279.6 ng/L) and anomaly of the ST segment were observed [21]. Patel *et al*. [30] reported 5 male patients aged 24.6 ± 7.3 years with myocarditis diagnosed by CMR within 72 hours 1.8 ± 0.44 days after inoculation with mRNA COVID-19 vaccine. It was found that the incidence of pericarditis in older men is more common [6, 7]. To sum up, we found that myocarditis caused by the COVID-19 vaccine tends to occur in adolescents [35, 54, 55], mainly in men. In female patients, myocarditis with pericardial effusion is the most common finding. Most patients with myocarditis have mild symptoms and a good prognosis. The incidence of myocarditis mainly occurred after the second dose of vaccine injection, and the incidence associated with the first and third doses was relatively low.

## 5. Pathogenesis of Myocarditis Caused by COVID-19 mRNA Vaccine

**Fig. 1.**
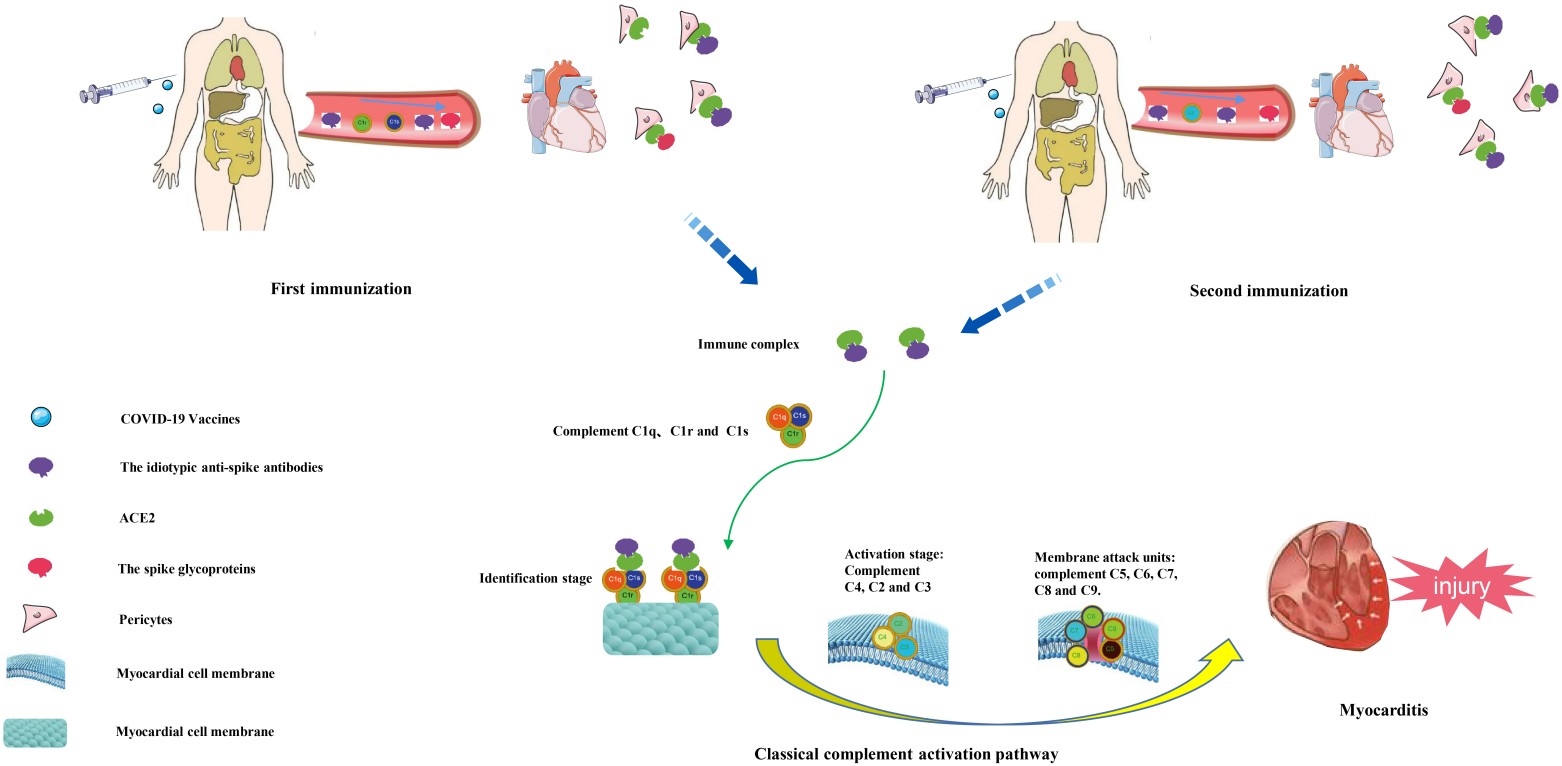
**Pathogenesis of myocarditis caused by human injection of COVID-19 vaccine**.

Vaccine inoculation has long been established to lead to myocarditis and cardiomyopathy. Morgan *et al*. [56] reported 21 patients with myocarditis after smallpox vaccination. Histopathological examination found monocytes were the main type of immune cell. The pathogenesis of myocarditis has been associated with the cross-reaction mechanism of susceptible individuals to stimulate the vaccine to cause an autoimmune response, this mechanism is considered to be multifactorial, and the pathogenesis is not clear, although it is widely believed that immune-mediated mechanisms play an important role [57]. Although myocarditis caused by COVID-19 has been widely reported, the underlying mechanism remains unclear. Current evidence suggests that the interaction between COVID-19 spike protein and autoantibodies is involved in the pathogenesis of myocarditis. It has been shown that the antibodies of COVID-19 spike protein and human peptide proteins such as α-myosin can interact with each other [15]. Bozkurt *et al*. [15] advocated that the important mechanism of myocarditis is the spike glycoprotein, and the human peptide protein sequence of SARS-CoV-2 (for example: α-Myosin) could cross-react, which is a molecular simulation reaction between spike glycoprotein and its antigen. The spike glycoprotein can cause the secretion of interferon and other factors and eventually lead to the immune response of multiple organs, thus causing myocarditis [58]. It is widely thought that in individuals with genetic susceptibility, the spike glycoprotein may lead to dysregulation of the original pathway, and finally lead to activation of the immune pathway and inflammatory response [59]. In recent months, much emphasis has been placed on elucidating the pathogenesis of myocarditis caused by the COVID-19 mRNA vaccine. It has been reported that the COVID-19 mRNA vaccine consists of a series of the mRNA-lipid nanoparticle protein [7, 60, 61]. The pathogenesis of myocarditis may depend on the cross-reaction mechanism in susceptible individuals, which leads to an autoimmune response, and this mechanism is considered to be multifactorial [57]. Interestingly, mRNA can activate the immune system, and part of these immune pathways can play an inflammatory response in some special individuals, resulting in myocarditis [59]. Bozkurt *et al*. [15] believed that the COVID-19 mRNA vaccine did not cause new immune-mediated reactions since, in some individuals, the preexisting dysregulated pathways are triggered, causing an increase in clonal B cells, immune complexes, and inflammation. Katalin *et al*. [62] advocated that compared with the modified mRNA, the expression ability of dendritic cell RNA may be reduced since dendritic cells in these susceptible individuals express cytokines, which can activate immune markers leading to decreased ability to suppress immunogenic immunity. Studies have shown that the modified nucleoside components of the COVID-19 mRNA vaccine can inhibit the body’s innate immunity [62]. However, the autoimmune response may not be reduced in susceptible individuals, leading to abnormal immune responses [59, 62]. COVID-19 mRNA vaccine prevents the binding of S protein to ACE2 and produces cellular and humoral immunity by using nucleoside-modified mRNA encoding the virus membrane s glycoprotein and producing antibodies to S protein [15]. In addition, Bozkurt *et al*. [15] reported a case of myocarditis caused by the COVID-19 mRNA vaccine, whereby the number of natural killer (NK) cells doubled. It is widely acknowledged that NK cells can destroy cells infected or rejected by the virus, and the proliferation of NK cells may be the mechanism leading to pathological immune response or adaptive response of myocarditis. Nonetheless, further experiments are required to validate this finding [15]. Given that excipients of the COVID-19 mRNA vaccine may account for the hypersensitivity of some receptors, hypersensitivity may also be one of the important causes of myocarditis. Kadkhoda [63] reported that after the injection of COVID-19 vaccine, the human body will produce idiotypic anti-spike antibodies, especially after the second dose of vaccine, the amount of antibody will be more. The idiotypic anti-spike antibody can simulate the expression of spike glycoproteins and combine with ACE2 receptor on the surface of cardiomyocytes to form a fixed immune complex. The immune complex can activate the complement system through the classical pathway and ultimately lead to inflammation or damage of cardiomyocytes (Fig. 1). In conclusion, myocarditis caused by the COVID-19 mRNA vaccine is currently considered multifactorial [57, 64]. Future studies will focus on better understanding mRNA expression and autoimmune mechanisms and pathways.

After injection of COVID-19 vaccine into human body, one idiotypic anti-spike antibody will be produced in the body. The idiotypic anti-spike antibody can come to the heart together with the spike glycoprotein through blood circulation. The idiotypic anti-spike antibody can simulate the expression of spike glycoprotein and combine with cardiac ACE2 receptor to form an immune complex. Immune complexes can activate the complement system through the classical pathway of complement. First, they bind to C1q, C1r and C1s and recognize the cardiomyocyte membrane, and then activate C4, C2 and C3 to activate the cardiomyocyte membrane. Finally, C5, C6, C7, C8, and C9 attack the cell membrane, eventually destroying the cardiomyocyte membrane and causing myocardial damage.

## 6. Suggestions on the Evaluation of COVID-19 mRNA Vaccine-Induced Myocarditis 

Herbs are widely used to treat and prevent various infectious diseases. Abiri *et al*. [65] discussed the mechanism of action of active compounds extracted from plants in the treatment of COVID-19, while the studies related to herbal medications are small and usually not randomized controlled trials (RCTs). However, the COVID-19 mRNA vaccine plays an active role in preventing coronavirus infection. This article focuses on the COVID-19 mRNA vaccine-induced myocarditis and puts forward the following suggestions. Myocarditis caused by the COVID-19 mRNA vaccine tends to occur in adolescents and men. In cases with chest pain, fever, and other symptoms after vaccination, emphasis should be placed on ruling out myocarditis [19, 46]. In addition, myocarditis combined with pericardial effusion should be suspected in female patients with the above symptoms after vaccination [22]. Given that pericarditis tends to occur in older men, more emphasis should be placed on increasing awareness and prevention when obtaining written informed consent [6, 7, 66]. It has been established that myocarditis mostly occurs after the second dose of vaccine injection. Accordingly, patient education on myocarditis should be prioritized when injecting the second dose of vaccine, especially in people with myocardial disease history. In certain cases, extending the interval between the first and first doses should be considered to reduce the risk of adverse events [19, 20]. CMR has significant value for the early diagnosis of myocarditis caused by the COVID-19 mRNA vaccine. CMR and cardiac examinations should be conducted as soon as possible to diagnose cases presenting with chest discomfort and other symptoms after vaccination. In general, most patients with myocarditis caused by the COVID-19 mRNA vaccine have mild symptoms and should be actively treated at the onset, resulting in a good prognosis.

## 7. Conclusions

Although the immunogenicity of the COVID-19 mRNA vaccine has brought a series of diseases such as myocarditis to humans, the incidence is relatively low. Indeed, immunization with the vaccine has significantly reduced the incidence rate and severe mortality of COVID-19 worldwide. As a non-invasive diagnostic tool for diagnosing myocarditis caused by the COVID-19 mRNA vaccine, CMR can effectively evaluate the myocardial function and structure changes, complement the limitations of laboratory and pathological examination during the clinical diagnosis process, and understand the health status of patients through long-term follow-up examination of CMR. By studying the pathogenesis and influencing factors of myocarditis caused by the COVID-19 mRNA vaccine, we can optimize and improve the vaccination program to efficiently reduce adverse events.
